# Supplementary material for: Polyketide synthase gene clusters in the ericoid mycorrhizal fungus Oidiodendron maius: genome-wide prediction, expression profiling, and functional characterization of knockout mutants
Source: Front Plant Sci. 2026 Sep 16;17:1924430. doi: 10.3389/fpls.2026.1924430 (PMC13623903; doi:10.3389/fpls.2026.1924430)
Supplement: Supplementary file 1 [file SupplementaryFile1.zip › Supplementary Materials/Supplementary Folder 1.zip!/Oidma1_AssemblyScaffolds_Repeatmasked/knownclusterblast/cluster29/jgi.p_Oidma1_44030_mibig_hits.html]

| MIBiG Protein | Description | MIBiG Cluster | MiBiG Product | % ID | % Coverage | BLAST Score | E-value |
| --- | --- | --- | --- | --- | --- | --- | --- |
| CCA54216.1 | Aldo-keto\_reductase | BGC0000893 | Chloramphenicol\_biosynthetic\_gene\_cluster | 44 | 88.9880952381 | 235 | 1.2e-61 |
| ADE34525.1 | SsfF | BGC0000269 | SF2575\_biosynthetic\_gene\_cluster | 41 | 96.130952381 | 233 | 3.4e-61 |
| EFG07997.1 | Aldo/keto\_reductase | BGC0000843 | Clavams\_biosynthetic\_gene\_cluster | 41 | 94.3452380952 | 224 | 2.1e-58 |
| AFH74311.1 | Cvm1-like\_aldo/keto\_reductase | BGC0001151 | Clavams\_biosynthetic\_gene\_cluster | 39 | 94.3452380952 | 209 | 7e-54 |
| WP\_029025949.1 | hypothetical\_protein | BGC0000198 | Arenimycin\_B\_biosynthetic\_gene\_cluster | 37 | 94.6428571429 | 183 | 3.1e-46 |
| ANC94984.1 | AlmCI | BGC0001396 | Aldgamycin\_biosynthetic\_gene\_cluster | 30 | 88.9880952381 | 127 | 3.5e-29 |
| ACX83630.1 | putative\_aldo/keto\_reductase | BGC0000221 | Erdacin\_biosynthetic\_gene\_cluster | 31 | 97.0238095238 | 125 | 1.3e-28 |
| ABW91156.1 | 4-keto-6-deoxy\_NDP-hexose\_2,3-reductase | BGC0000019 | Angolamycin\_biosynthetic\_gene\_cluster | 29 | 92.8571428571 | 124 | 2.9e-28 |
| ABB52534.1 | putative\_oxidoreductase | BGC0000047 | Dihydrochalcomycin\_biosynthetic\_gene\_cluster | 30 | 88.9880952381 | 122 | 8.6e-28 |
| ABB05109.1 | LipDig3 | BGC0001003 | Lipomycin\_biosynthetic\_gene\_cluster | 29 | 93.75 | 116 | 4.7e-26 |
| AAS90055.1 | NorA | BGC0000009 | Aflatoxin\_biosynthetic\_gene\_cluster | 27 | 93.75 | 116 | 6.1e-26 |
| ACB37756.1 | putative\_oxidoreductase | BGC0000162 | Tetrocarcin\_A\_biosynthetic\_gene\_cluster | 30 | 89.880952381 | 104 | 2.4e-22 |
